# Lactate-utilizing community is associated with gut microbiota dysbiosis in colicky infants

**DOI:** 10.1038/s41598-017-11509-1

**Published:** 2017-09-11

**Authors:** Van T. Pham, Christophe Lacroix, Christian P. Braegger, Christophe Chassard

**Affiliations:** 1 Laboratory of Food Biotechnology, Institute of Food, Nutrition and Health, ETH Zurich, Zurich, Switzerland; 2 Division of Gastroenterology and Nutrition, University Children’s Hospital Zurich, Zurich, Switzerland; 3 Université Clermont Auvergne, INRA, UMRF, F-1500 Aurillac, France

## Abstract

The aetiology of colic, a functional gastrointestinal disorder in infants, is not yet resolved. Different mechanisms have been suggested involving the gut microbiota and intermediate metabolites such as lactate. Lactate can be metabolized by lactate-utilizing bacteria (LUB) to form different end-products. Using a functional approach, we hypothesized that H_2_ production and accumulation by LUB is associated with the development of colic. The LUB communities in the feces of forty infants, including eight colicky infants, were characterized using a combination of culture- and molecular-based methods, and metabolite concentrations were measured by HPLC. Interactions among LUB strains isolated from feces were investigated with pure and mixed cultures using anaerobic techniques. We emphasized high prevalence of crying, flatulence, colic and positive correlations thereof in the first 3 months of life. Crying infants showed significantly higher ratio of LUB non-sulfate-reducing bacteria (LUB non-SRB) (H_2_-producer), to LUB SRB (H_2_-utilizer) at 3 months. Colicky infants had significantly higher number of H_2_-producing *Eubacterium hallii* at 2 weeks compared to non-colicky infants. We revealed the function of *Desulfovibrio piger* and *Eubacterium limosum* to reduce H_2_ accumulation in co-cultures with H_2_-producing *Veillonella ratti*. Our data suggest that the balance between H_2_-producing and H_2_-utilizing LUB might contribute to colic symptoms.

## Introduction

Colonization of the neonatal gut is one of the most important biological events in one’s life. The establishment of different bacterial groups and their metabolic outcomes are crucial for not only early life developments but also potentially for long term health^1–3^. Recently, microbes have been detected in the intrauterine environment such as the amniotic fluid, umbilical cord blood, fetal membranes, placenta, and meconium, which questions the generally accepted concept of a sterile fetal gastrointestinal tract^1, 4, 5^. Regardless of intrauterine exposure, the greatest influence on the development and establishment of gut microbiota occurs likely at birth, when the infant is exposed to vaginal, fecal, and skin microbiota from the mother^6–8^. Another postnatal route of mother-infant microbial exchange that promotes the colonization and maturation of the infant gut microbiota is maternal breast milk, which provides a wide range of commensal and beneficial microbes including *Bifidobacterium* and *Lactobacillus* ^9–11^. Moreover, human milk oligosaccharides function as prebiotics by supporting the growth of such beneficial bacteria^12^.

Within the first week of life, bacterial groups such as *Staphylococcus*, *Enterococcus*, *Streptococcus*, and members of the *Enterobacteriaceae* reach high population densities that subsequently create a reduced environment that allows the establishment of strict anaerobes^13^. In the last decades, many studies investigated factors that could alter this colonization period, with the most robust evidence pointing toward mode of delivery, mode of feeding, and use of antibiotics^3^. The development of high throughput sequencing techniques has allowed further important insights into the overall composition, diversity, and the shift of gut microbiota across age^13–18^. During the first three years of life, the infant gut microbiota evolves towards the bacterial composition and diversity found in adults^19, 20^.

Because most primary colonizers in the infant gut are lactate-producing bacteria (LPB), lactate must be efficiently reused to avoid potential negative consequences of lactate accumulation. The accumulation of lactate could lead to detrimental consequences such as acidosis, neurotoxicity, and cardiac arrhythmia^21^. However, excess H_2_ production from lactate utilization may be responsible for high incidence of acute bloating and cramping in early life^22^. Furthermore, increased sulfate-reducing bacteria (SRB) numbers could result in elevated hydrogen sulfide (H_2_S) levels, which could potentially cause colonic pain and gastrointestinal discomfort^23, 24^.

One of the important functions of the gut microbiota is to salvage nutrients and energy by fermentation. This involves metabolic cross-feeding, where metabolites produced by one species serve as substrates for other species^25^. Cross-feeding of lactate in infants was recently demonstrated in a cohort study of 40 Swiss infants^26^. In this study, there were significant positive correlations between LPB and lactate-utilizing bacteria (LUB). Among the LUB community, H_2_-producing *Veillonella* were identified as one of the keystone genera. Other LUB were also identified, such as the butyrate-producer *Eubacterium hallii*, which also produces H_2_, or SRB that produce H_2_S. In a recent Brazilian cohort study of 12 infants, lactate-utilizing, butyrate-producing *E. limosum* were detected in fecal samples of four infants, suggesting the colonization of this genus in the first year of life^27^. Interestingly, metabolic cross-feeding of H_2_ was demonstrated within the LUB community in the Swiss cohort, where H_2_ produced by *Veillonella* and *E. hallii* serves as a preferable substrate for SRB like *Desulfovibrio piger* ^26^. It is therefore important to develop a mechanistic understanding of the complex interactions of bacterial species within the LUB community, and investigate the role of key players and metabolic balance on infant gut health.

While dysbiosis of the adult gut microbiota has been linked to a wide range of diseases, including IBD, obesity, and colon cancer^28^, little is known about the role of infant gut microbiota in gastrointestinal diseases. Infantile colic (IC) is a functional gastrointestinal disorder that affects up to 20% of infants, regardless of their mode of feeding^29, 30^. Despite the self-limiting nature of the condition, IC has psychological and economical ramifications for parents, and to some extent, to the health care system^31^. Long-term consequences for infants have also been identified, such as increased risk of anxiety, aggression, hyperactivity, and allergy^32, 33^. In the past decades, interesting psychosocial and physiological hypotheses regarding etiology of IC have been suggested^34^. Recently, studies have focused on the role of infant gut microbiota in IC, albeit no consensus was reached. Distinct gut microbial signatures between colicky and non-colicky infants were reported in several studies. Savino *et al*. showed that breast-fed colicky infants had fewer lactobacilli and more gram-negative anaerobic bacteria compared to non-colicky infants^35^. A recent study using a microarray technique showed that a colic phenotype correlated positively with specific groups of Proteobacteria, including *Escherichia*, *Klebsiella*, *Serratia*, *Vibrio*, and *Pseudomonas*, but negatively with Bacteroidetes and Firmicutes phyla in the first weeks of life^36^. A less diverse fecal microbiota was also observed in infants with colic^36, 37^. On the other hand, no differences in the gut microbiota composition were found between the colicky infants at the time of colic and the controls in another study^38^. To our knowledge, there is no microbe or microbial group that can be specifically associated with IC. Furthermore, no robust treatment for IC is currently available.

In the present study, we hypothesized that the metabolism of lactate, the intermediate product of carbohydrate metabolism, plays a key role in the etiology of gastrointestinal symptoms such as IC in infants. The metabolic impact of microbiota on gut health could be mediated by the accumulation of either lactate, or other end products of the lactate utilization process, such as H_2_ or H_2_S. We compared levels of functional bacterial groups involved in lactate and H_2_ metabolism in colicky and non-colicky infants. Furthermore, isolated strains representing the key lactate-utilizing species in the infant gut were selected to investigate their interactions in co-cultures by *in vitro* fermentation experiments under strict anaerobic conditions.

## Results

### Baseline and gastrointestinal characteristics in infant cohort from birth to 6 months

The mean gestational age of our cohort (n = 40) was 40 weeks (ranging from 37 to 42 weeks) (Table 1). The population comprised of 55% female and 45% male. Eleven infants (27.5%) were born by caesarean delivery. Within our cohort, there was a set of dizygotic twins, with one non-colicky infant and one that was diagnosed with IC at 2 months old.

**Table 1:** General and gastrointestinal characteristics of the study population (n = 40 infants).

General characteristics					
**Gestation (weeks)**					
37	2 (5.0)				
38	3 (7.5)				
39	11 (27.5)				
40	13 (32.5)				
41	9 (22.5)				
42	2 (5.0)				
**Mode of delivery**					
Caesarean section	11 (27.5)				
Vaginal delivery	29 (72.5)				
**Gender**					
Female	22 (55.0)				
Male	18 (45.0)				
**Birth weight (kg)**					
>4	3 (7.5)				
3.5–4	13 (32.5)				
3–3.5	18 (45.0)				
2.5–3	4 (10.0)				
<2.5	2 (5.0)				
**Gastrointestinal symptoms**	**2 weeks**	**1 month**	**2 months**	**3 months**	**6 months**
**Colic Diagnostic Criteria 1** ^**a**^					
No	32 (80.0)	21 (53.9)	26 (68.4)	32 (88.9)	39 (97.5)
Yes	8 (20.0)	18 (46.1)	12 (31.6)	4 (11.1)	1 (2.5)
**Colic Diagnostic Criteria 2** ^**b**^					
No	37 (92.5)	37 (94.9)	34 (89.5)	36 (100)	40 (100)
Yes	3 (7.5)	2 (5.1)	4 (10.5)	0 (0)	0 (0)
**Colic Diagnostic Criteria 3** ^**c**^					
No	0 (0)	0 (0)	0 (0)	0 (0)	0 (0)
Yes	40 (100)	39 (100)	38 (100)	40 (100)	40 (100)
**Colic episode**					
No	37 (92.5)	37 (94.9)	34 (89.5)	36 (100)	40 (100)
Yes	3 (7.5)	2 (5.1)	4 (10.5)	0 (0)	0 (0)
**Colic**					
No	32 (80)	NA	NA	NA	NA
Yes	8 (20)	NA	NA	NA	NA
**Flatulence/Bloating**					
No	17 (42.5)	11 (28.2)	12 (31.58)	20 (55.6)	35 (87.5)
Yes	23 (57.5)	28 (71.8)	26(68.4)	16 (44.4)	5 (12.5)
Light	14 (35)	10 (25.6)	10 (26.3)	11 (30.5)	4 (10)
Mild	7 (17.5)	15 (38.5)	12 (31.6)	5 (13.9)	1 (2.5)
Strong	2 (5.0)	3 (7.7)	4 (10.5)	0 (0)	0 (0)
**Cramp**					
No	20 (50.0)	14 (35.9)	12 (31.6)	25 (69.4)	33 (82.5)
Yes	20 (50.0)	25 (64.1)	26 (68.4)	11 (30.6)	7 (17.5)
Light	11 (27.5)	9 (23.1)	13 (34.29	6 (16.7)	7 (17.5)
Mild	7 (17.5)	15 (38.5)	12 (31.6)	5 (13.9)	0 (0)
Strong	2 (5.0)	1 (2.6)	1 (2.6)	0 (0)	0 (0)
**Crying**					
<1 h/d	16 (42.1)	9 (23.7)	13 (34.2)	16 (44.4)	29 (72.5)
>1 h/d	22 (57.9)	29 (76.3)	25 (65.8)	20 (55.6)	11 (27.5)

Data were presented as n(%).

^a^Paroxysms of irritability, fussing, or crying that start and stop without obvious cause;

^b^Episodes lasting 3 or more hours per day and occurring at least 3 days per week for at least 1 week;

^c^No failure to thrive.

Data were presented as n(%).

**Figure 1:**
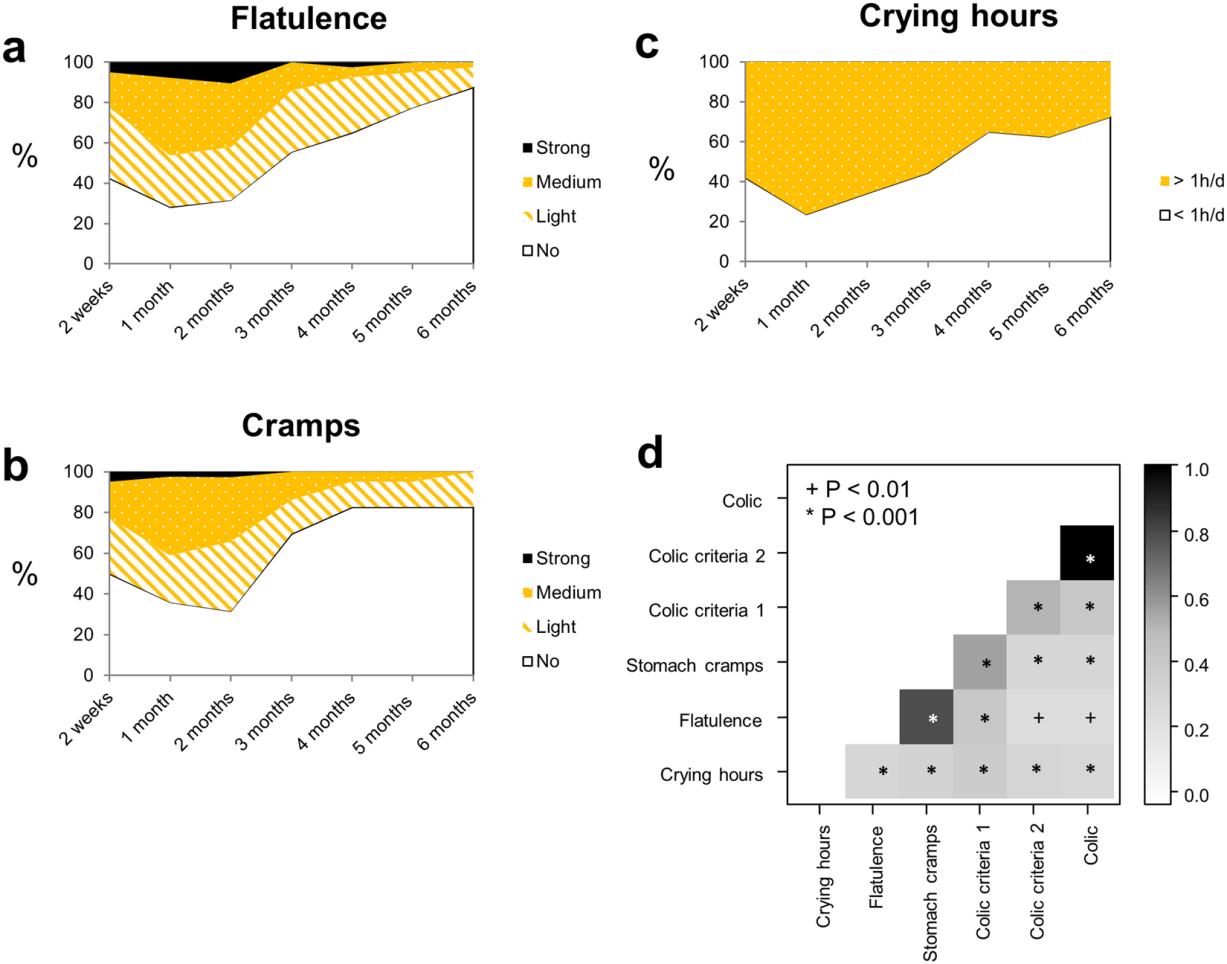
Prevalence and correlations of infant gastrointestinal symptoms in the first 6 months of life. Prevalence of flatulence (**a**), stomach cramps (**b**), and crying hours (**c**) of infants from 2 weeks to 6 months (n = 40); >1h/d: infants who cried more than 1 h/d; <1h/d: infants who cried less than 1 h/d. (**d**) Spearman pairwise correlation map of infant gastrointestinal symptoms during the first 3 months of life (n = 150). The color gradient denotes Spearman R value. Colic criteria 1: paroxysms of irritability, fussing, or crying that start and stop without obvious cause; Colic criteria 2: episodes lasting 3 or more hours per day and occurring at least 3 days per week for at least 1 week; Colic: infants diagnosed by Rome III criteria (see Methods).

Eight infants (20%) fulfilled Rome III diagnostic criteria for IC, two at 2 weeks, one at 1 month, four at 2 months, and one at both 2 weeks and 1 month (Table 1). The number of infants suffering from paroxysms of irritability, fussing, or crying that start and stop without obvious cause (Colic criteria 1) were 8 (20%), 18 (46.2%), 12 (31.6%), 4 (11.1%), and 1 (2.5%) at 2 weeks, 1 month, 2, 3, and 6 months, respectively. The number of infants had such episodes lasting 3 or more hours per day and occurring at least 3 days per week for at least 1 week (Colic criteria 2) were 3 (7.5%), 2 (5.1%), and 4 (10.5%) at 2 weeks, 1 month, and 2 months, respectively. All infants had no failure to thrive (Colic criteria 3). Flatulence was reported in 57.5% of infants at 2 weeks, and prevalence gradually increased at 1 month (71.8%) and 2 months (68.4), and declined at 3 months (44.4%) and 6 months (12.5%) (Fig. 1a). At 2 weeks, half of the population (n = 20) experienced stomach cramps. The prevalence of cramps peaked at 1 month (64.1%) and 2 months (68.4%), and decreased at 3 (30.6%) and 6 months (17.5%) (Fig. 1b). We stratified crying infants into two groups: infants who cried more than 1 h/d, and infants who cried less than 1 h/d. The prevalence in the former group was 57.9%, 76.3%, 65.8%, 55.6%, and 27.5% at 2 weeks, 1 month, 2, 3, and 6 months, respectively (Fig. 1c).

Spearman’s correlations between crying hours, flatulence, stomach cramps, colic criteria, and Rome III colic were investigated by pooling data from the first 3 months of life (n = 150), when gastrointestinal symptoms were most prevalent. Positive correlations were found between crying hours and flatulence, stomach cramps, and Rome III colic (*q* < 0.001) (Fig. 1d). Flatulence was strongly correlated with cramps and colic criteria 1 (*q* < 0.001).

Taken together, our data emphasize the high prevalence of crying, flatulence, and IC, and their positive correlations in the first 3 months of life.

### Lactate-utilizing bacteria in infants with and without gastrointestinal discomforts

**Figure 2:**
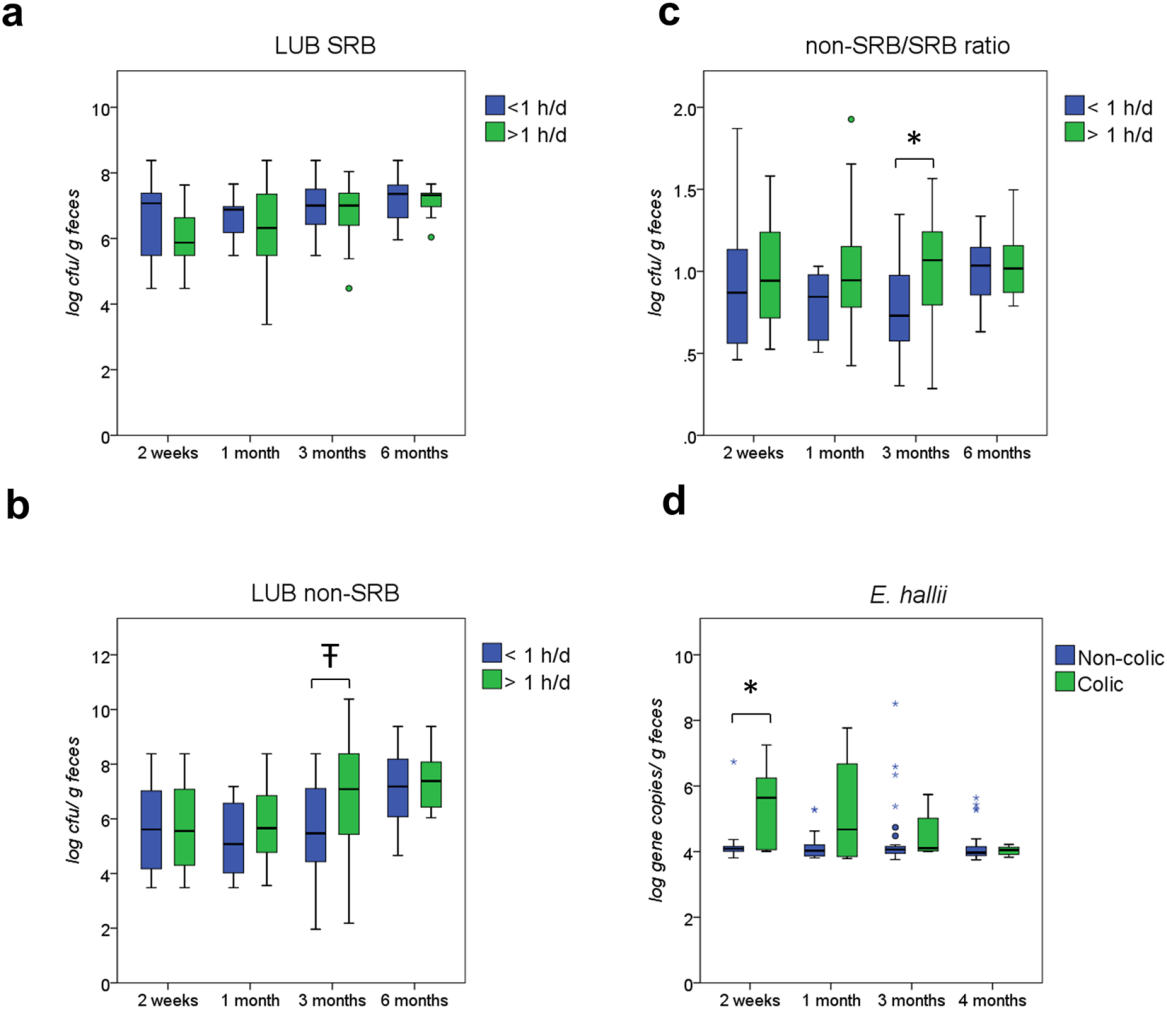
(**a**–**c**) Total counts of LUB SRB, LUB non-SRB, and LUB non-SRB/LUB SRB ratio in feces of infant crying <1 h/d and infant crying >1 h/d at 2 weeks, 1 month, 3, and 6 months. (**d**) *E. hallii* levels between colicky (n = 8) and non-colicky (n = 32) infants at 2 weeks, 1 month, 3, and 6 months. Colic: infants diagnosed by Rome III criteria (see Methods). Central horizontal line shows the median, upper and lower box border show the 90th and 10th centile respectively, upper and lower whisker show the 95th and 5th centile respectively. Open circle designates outliers; asterisk designates extreme values using a step of 1.5 x IQR (interquartile range). *P < 0.05; Ŧ *P = *0.056; LUB, lactate-utilizing bacteria; SRB, sulfate-reducing bacteria.

Colonization of LUB was investigated by analysing infant feces using traditional culture-based methods and qPCR. The culturable LUB community consisted of lactate-utilizing sulfate-reducing bacteria (LUB SRB) and lactate-utilizing non-sulfate-reducing bacteria (LUB non-SRB). The latter group consists mainly of H_2_-producing bacteria, such as *Veillonella* and *E. hallii*. Infants who cried more than 1 h/d harbored higher levels of LUB non-SRB at 1 month and 3 months (*P* = 0.056) than infants who cried less than 1 h/d (Fig. 2b) (see Supplementary Table S1). A significantly lower non-SRB/ SRB ratio was detected at 3 months (*P* < 0.05) in infants who cried less than 1 h/d (Fig. 2c).

A large inter-individual variability was observed for taxonomic abundance in feces of infants with gastrointestinal symptoms (see Supplementary Fig. S1b). We compared LUB levels between infants with and without IC by culture-based and qPCR methods (see Supplementary Table S1). Colicky infants had significantly higher numbers of *E. hallii* at 2 weeks compared to non-colicky infants (5.39 ± 1.23 vs. 4.16 ± 0.49 log copies/g feces, *P* < 0.05) (Fig. 2d). Moreover, H_2_-utilizing SRB were not detected by qPCR in colicky infants at 2 weeks. At 1 month and 3 months, SRB were detected in 33.3% and 16.7% colicky infants. Our data suggest that crying and colicky infants have higher lactate-utilizing H_2_-producing bacteria.

Levels of LUB in feces of the dizygotic twins in the cohort were compared by using culture-based, qPCR and MiSeq sequencing methods. The colicky infant had a higher level of LUB non-SRB (6.7 vs. 3.9 log cfu/g feces) and a lower level of LUB SRB at 2 weeks (5.5 vs. 8.4 log cfu/g feces) compared to the non-colicky twin in the pair (see Supplementary Fig. S2a). *Veillonella* numbers detected by qPCR were also higher in the colicky infant at 3 months compared to the non-colicky infant (7.4 vs. 4.8 log copies/g feces) (see Supplementary Fig. S2b). No other differences in bacterial levels detected by qPCR were observed between the twins (data not shown). Fecal samples from the twins were also compared using multiplex sequencing of 16 S rRNA genes with Illumina high-throughput sequencing. The results confirmed qPCR data, with a higher relative abundance of *Veillonella* in the colicky infant feces at 2 (19% vs. 1%) and 3 months (19% vs. 2%) (see Supplementary Fig. S2c).

### Metabolic activity in colicky and non-colicky infants

Glucose, lactate and short chain fatty acids (SCFA) concentrations measured by HPLC in fecal water extracted from 8 colicky and 32 non-colicky infants at 2 weeks, 1 month, 2, 3, and 6 months revealed large inter-individual variabilty in both groups at all time points (see Supplementary Table S2). There was no difference in metabolic profiles between colicky and non-colicky groups.

Nonetheless, our data revealed different metabolic profiles between the dizygotic twins. The colicky infant showed 2-fold higher fecal lactate concentrations at 2 and 3 months (101.9 mM and 43.0 mM, respectively) compared to the non-colicky infant (40.7 mM and 21.8 mM, respectively) (see Supplementary Fig. S2d). In contrast, high formate concentrations were detected in fecal samples of the non-colicky infant at 2 weeks, 2, and 3 months (89.9 mM, 176.8 mM, and 151.1 mM, respectively) compared to no formate detected in the feces of the colicky infant. Concentrations of acetate (70.2 vs. 42.1 mM), propionate (18.5 vs. 0.1 mM), and butyrate (9.9 vs. 0.0 mM) were also higher in the non-colicky infant compared to the colicky infant at 3 months.

### Characterization of lactate-utilizing bacteria isolated from infant feces

**Figure 3:**
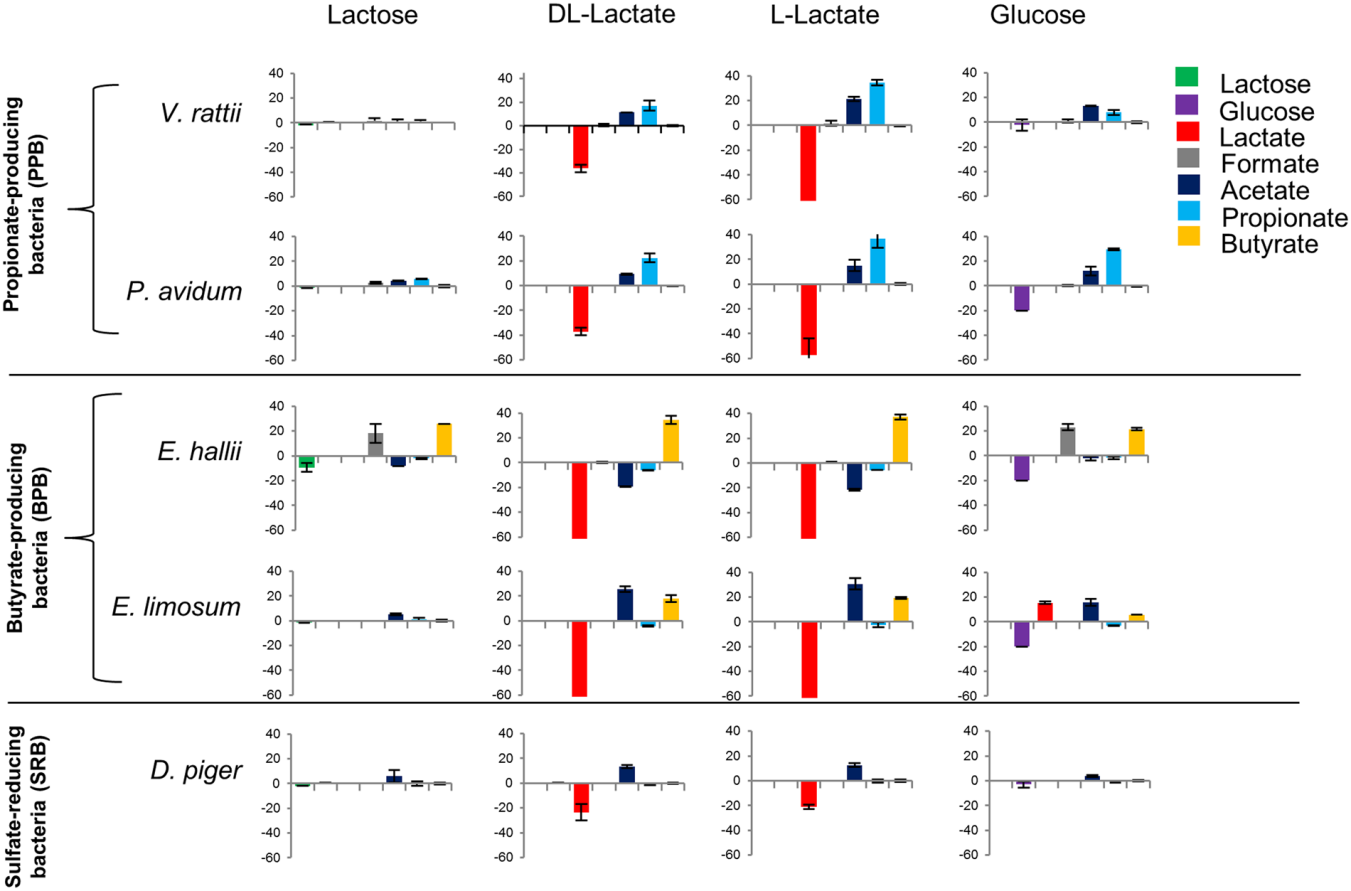
Consumption of lactose, glucose, and lactate and production of formate, acetate, propionate, and butyrate of LUB after 48 h incubation in YCFA medium supplied with lactose, DL-lactate, L-lactate, and glucose. Positive and negative values indicate production and consumption, respectively.

Using YCFA medium containing lactate as the sole carbon source, we isolated *Propionibacterium avidum* and *Veillonella ratti* from feces of infants at 2 weeks, and *Eubacterium limosum* from feces of a 5-month-old infant. From six colicky and seven non-colicky infants, *E. limosum* and *P. avidum* were isolated from non-colicky infants while *V. ratti* were isolated in both colicky and non-colicky infants. Isolated strains of *E. limosum*, *P. avidum*, and *V. ratti*, together with two other LUB, *E. hallii* (DSM 3353) and *D. piger* (DSM 749), were characterized by their ability to use different substrates, including lactose, DL-lactate, L-lactate, and glucose, to produce SCFA. Figure 3 shows the metabolites produced by pure cultures of two propionate-producing LUB strains (P-LUB; *V. ratti* and *P. avidum*), two butyrate-producing LUB strains (B-LUB; *E. hallii* and *E. limosum*); and one LUB SRB strain (*D. piger*) in YCFA medium supplemented with lactose, DL-lactate, L-lactate or glucose as a sole carbon source. H_2_ production and OD measurements of these pure cultures are shown in Supplementary Figs S3 and S4, respectively.

P-LUB strains metabolized half of the lactate added to the DL-lactate medium, whereas L-lactate was consumed completely in L-lactate medium. Among the strains of LUB that were tested, *V. ratti* showed the fastest growth in DL- and L-lactate medium, reaching OD_600_ levels of 0.5 and 0.4 after 8 h; and OD_600_ levels of 0.8 and 1.0 after 12 h, respectively (see Supplementary Fig. S4). Although both tested P-LUB showed growth in glucose medium, *P. avidum* was the only P-LUB strain that showed the consumption of glucose. This strain produced the highest OD_600_ in glucose medium (OD_600_ levels of 0.7 and 1.6 after 8 and 12 h incubation) compared to other media. Interestingly, in YCFA medium supplied with DL-lactate, *V. ratti* produced 19.48 ± 1.81% H_2_ while *P. avidum* did not produce H_2_ (see Supplementary Fig. S3). The ability to use glucose was also observed in two B-LUB strains, albeit via different metabolic pathways. *E. hallii* used 20 mM glucose to produce 21.2 mM formate and 22.3 mM butyrate. *E. limosum* used the same amount of glucose to produce 16.2 mM lactate, 13.6 mM acetate, and 5.9 mM butyrate. All B-LUB strains were able to utilize both D- and L-lactate. After 48 h, *E. limosum* consumed all DL- and L-lactate available (63.9 ± 0.0 mM and 66.5 ± 0.0 mM, respectively) and produced acetate (25.3 ± 2.2 mM and 30.6 ± 4.7 mM, respectively) and butyrate (17.9 ± 2.7 mM and 19.5 ± 0.8 mM, respectively). In the tested conditions, *D. piger* did not grow on media supplemented with lactose or glucose. It also showed limited metabolic capacity, converting one third of the total available DL- and L- lactate (23.7 mM ± 6.6 and 21.1 ± 1.6 mM, respectively) into acetate (13.5 ± 1.3 mM and 12.5 ± 1.6 mM, respectively). None of the tested strains was able to metabolize acetate and resistant starch (data not shown).

In conclusion, our data revealed distinct metabolic profiles for the five strains of LUB tested, including three strains isolated from infant feces.

### *In vitro* interactions between LUB strains

To investigate the interaction between LUB strains, *V. ratti* (P-LUB), *E. limosum* (B-LUB) and *D. piger* (LUB SRB) were chosen as representative of three lactate-utilizing pathways. *E. limosum* and *V. ratti* were chosen as representative LUB because they were isolate strains in fecal samples from our cohort by anaerobic roll tube method with medium containing lactate as sole carbon sourced. *D. piger* was chosen because it is the most common SRB in healthy adults^39^. Moreover, Hopkins *et al*. reported the detection of *Desulfovibrio* by qPCR in the feces of 8 infants from 0–6 months in a cohort study of 40 infants in the UK^40^. The three strains were grown in triplicate in 60 mM L-lactate YCFA medium as single and co-cultures.

**Figure 4:**
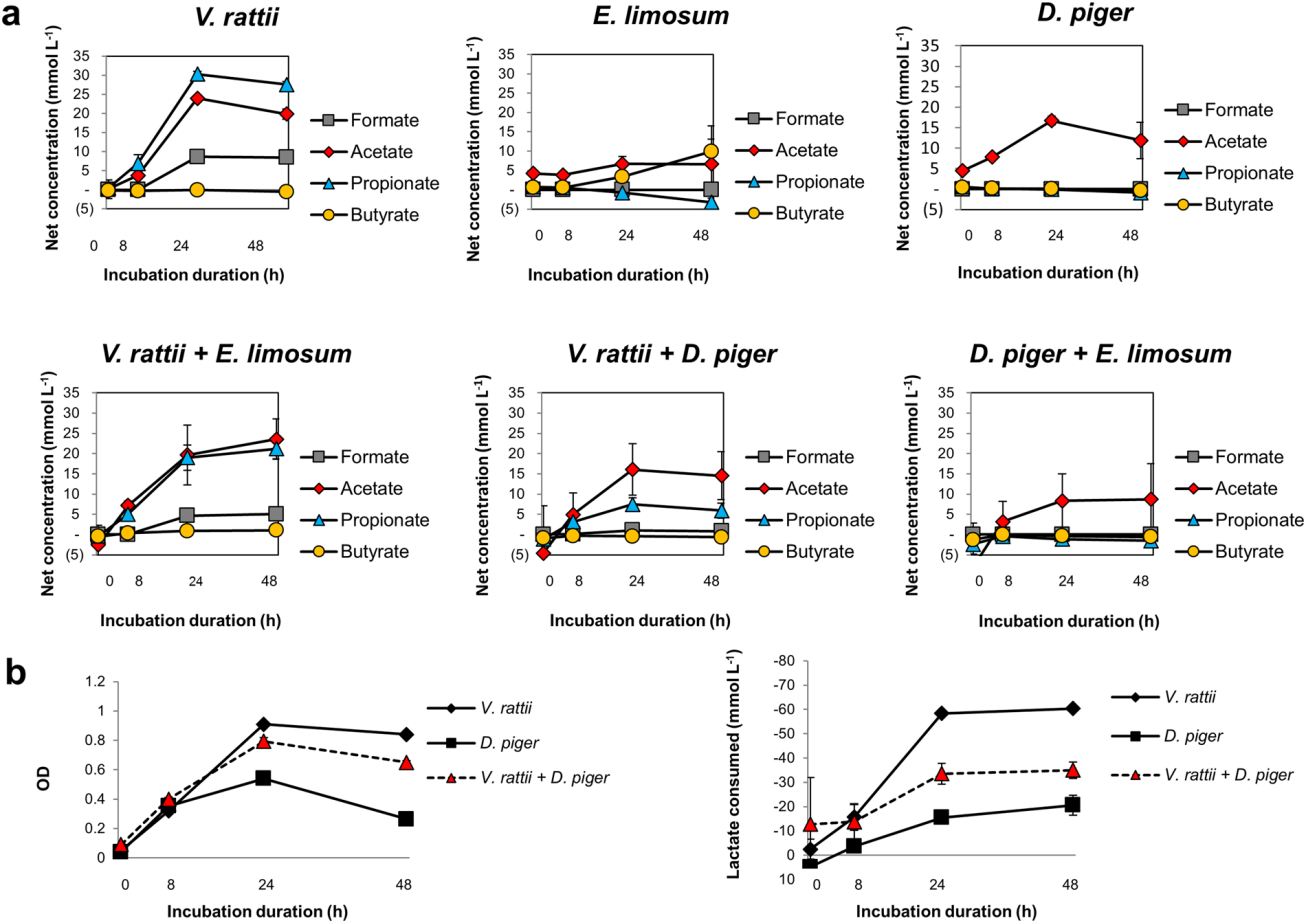
(**a**) Production of formate, acetate, propionate, and butyrate by single and co-culture of *V. ratti, E. limosum*, and *D. piger* (DSM 749) grown in triplicate in YCFA medium containing 60 mM DL-lactate. (**b**) OD_600_ and lactate consumption by single and co-culture of *V. ratti* and *D. piger*. *V. ratti* and *E. limosum* strains were isolated from infant feces^26^.

Figure 4a reports the metabolites produced by *V. ratti* and *E. limosum* in single and in co-cultures. *V. ratti* produced high amounts of both propionate (30.3 mM and 27.6 mM) and acetate (24.0 mM and 19.8 mM), but a lower amount of formate (8.6 mM and 8.5 mM) and no butyrate after 24 and 48 h, respectively. *E. limosum* produced only a small amount of butyrate; 3.4 ± 0.5 mM and 9.9 ± 3.2 mM after 24 and 48 h, respectively. However, no butyrate was detected during co-cultures of *E. limosum* with *V. ratti* and of *E. limosum* with *D. piger*. These results suggest high competitiveness of *V. ratti* and *D. piger* in co-cultures with *E. limosum*. These results were confirmed by qPCR data, by showing higher log copy numbers of *V. ratti* (10.94 ± 0.15) compared to *E. limosum* (8.78 ± 0.16) in *V. ratti* – *E. limosum* co-culture. The level of *D. piger* (8.62 ± 0.10) was 1 log higher compared to *E. limosum* (7.82 ± 0.10) in *D. piger* – *E. limosum* co-culture (see Supplementary Fig. S5a).

**Figure 5:**
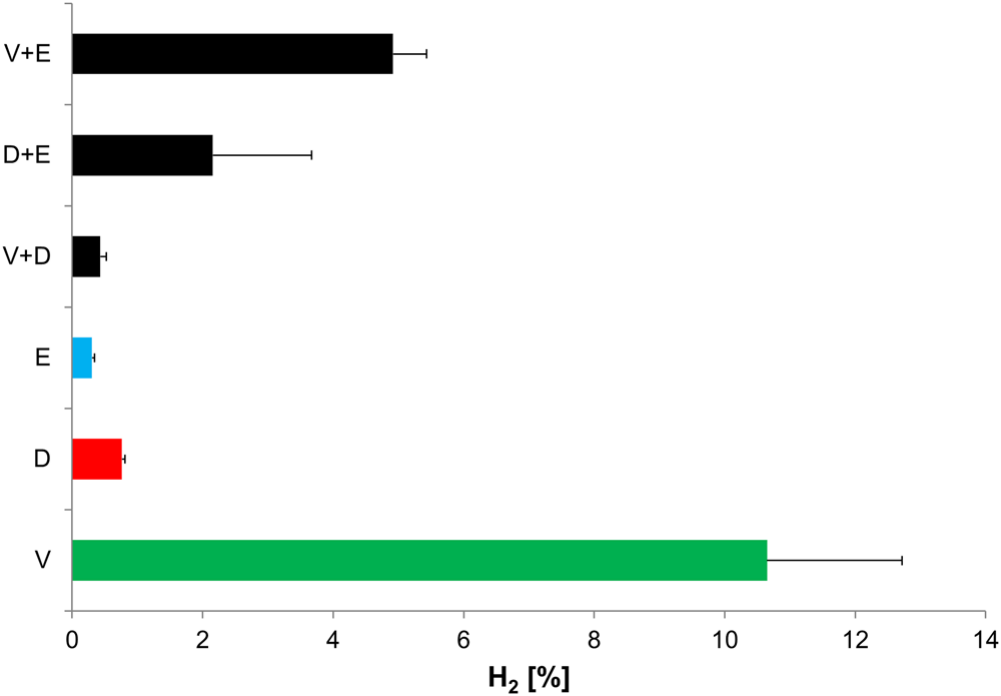
Production of H_2_ by *V. ratti* (V), *D. piger* (D), *E. limosum* (E) in single and co-cultures. Values are means ± SD (n = 2).

Interactions between *V. ratti* and *D. piger* were also investigated by testing metabolite production in single and co-cultures (Fig. 4a). In single cultures, *V. ratti* utilized lactate to produce propionate, acetate, and formate, while *D. piger* produced acetate as the sole metabolite (16.7 ± 1.1 mM and 11.9 ± 4.5 mM after 24 and 48 h, respectively). However, in co-cultures of *V. ratti* and *D. piger*, high acetate (16.1 ± 6.4 mM and 14.5 ± 5.9 mM) and low propionate amounts (7.4 ± 1.6 mM and 5.9 ± 1.8 mM) were produced after 24 and 48 h incubation, respectively. Furthermore, after 48 hours, the OD_600_ measured in co-cultures (0.65 ± 0.01) fell between the OD_600_ levels for the corresponding pure cultures (*V. ratti*: 0.84 ± 0.02; *D. piger*: 0.26 ± 0.01) (Fig. 4b). The same trend was observed for lactate consumption of co-cultures (34.99 ± 3.37 mM), which was intermediate between *V. ratti* (60.39 ± 0.39 mM) and *D. piger* (20.56 ± 4.07 mM) mono-cultures. Supplementary Fig. S5b shows a lower log gene copies increase of *V. ratti* in co-culture (1.66) compared to single-culture (3.21) after 48 h incubation. The log increase of *D. piger* remained unchanged in both co-cultures with *V. ratti* and *E. limosum*. Interestingly, when *V. ratti* was co-cultured with *D. piger* or *E. limosum*, the amount of H_2_ in the headspace of the tube decreased by 2- or 25-fold, respectively, compared to *V. ratti* single cultures (Fig. 5).

Using a simplified interaction model of three LUB belonging to different metabolic groups, our data provides insight into the metabolic interactions of predominant LUB strains in infant gut microbiota. Our data suggest the potential of *D. piger* and *E. limosum* to reduce H_2_ produced by *V. ratti* in co-culture.

## Discussion

Previous studies have indicated that primary colonizers of the infant gut, including *Lactobacillus*, *Bifidobacterium*, *Bacteroides*, *Streptococcus*, *Staphylococcus*, and *Enterococcus*, are LPB^13^. Therefore, large amounts of lactate are expected to be produced in the infant colon and lactate must be reused by LUB to prevent toxic accumulation. High prevalence and levels of LUB in the first 6 months of life have also been demonstrated^26^. In this study, we investigated the relationship of the LUB community to infant gastrointestinal symptoms in a cohort of 40 healthy infants, and elucidated supporting mechanisms by characterizing growth and metabolism of LUB strains in single and co-cultures. We detected specific LUB signatures for colicky and non-colicky infants and for infants crying more and less than 1 h/d. Our data suggest that an imbalance in the colonization of LUB H_2_-producing and -utilizing bacteria can lead to accumulation of H_2_, which contributes to a high prevalence of flatulence and colonic pain in the first months of life.

The role of gut microbiota in the pathogenesis of colic was recently raised and addressed in studies investigating the taxonomic differences between colicky and non-colicky infants. However, the lack of consensus suggests that molecular methods for taxonomic composition assessment is not sufficient to detect dysbiosis in colicky microbiota. Thus, complementary analysis is necessary, such as detecting functional microbial groups. Colic is characterized by short term crying of a few hours, suggesting temporal accumulation of metabolites. The main intermediate metabolite in the infant gut is lactate, which causes acidosis, neurotoxicity, and cardiac arrhythmia when it accumulates in the gut^21^. Hence, our approach was to investigate the role of bacterial groups utilizing lactate in colicky and crying in infants.

Among 40 infants in this study, eight were diagnosed with IC according to Rome III criteria^30^, representing 20% of the population. This prevalence is in agreement with previous studies^29^. In our study, colic episodes were detected exclusively within the first 2 months of life and resolved at 3 months, in concordance with other studies^41^. The stratification of crying infants into two groups, infants who cried more than 1 h/d and infants who cried less than 1 h/d, resulted in comparable numbers in both groups. Furthermore, with this stratification, we observed a common pattern between the prevalence of flatulence, stomach cramps, and crying hours, where highest prevalence was observed at 1 month and 2 months, and decreased over time. Interestingly, the same pattern was observed for breath H_2_ excretion^42, 43^. In a previous study, breath H_2_ excretion was significantly higher in infants with colic than those without^43^. Breath H_2_ of infants is produced mainly from fermentation of unabsorbed carbohydrate by the gut microbiota^43^. The high prevalence of flatulence, stomach cramps, crying hours, colic episodes, and H_2_ excretion exclusively within the first 2 months of life and their correlations suggests that this is an important time window when the metabolic production of gas can play a crucial role in infant gastrointestinal symptoms.

In this study, 3 months old infants crying more than 1 h/d had higher ratio of LUB non-SRB, which comprised the predominant H_2_-producing *Veillonella* and H_2_-producing *E. hallii*, to H_2_-utilizing LUB SRB. Furthermore, colicky infants harbored higher numbers of *E. hallii* at 2 weeks. There was no significant difference in metabolite concentrations between colicky and non-colicky infants, which could be explained by the remarkable inter-individual variability. Our results suggest that an increase in H_2_ production by LUB non-SRB and a decrease in H_2_ utilization by LUB SRB could lead to acute H_2_ accumulation associated with crying and IC.

**Figure 6:**
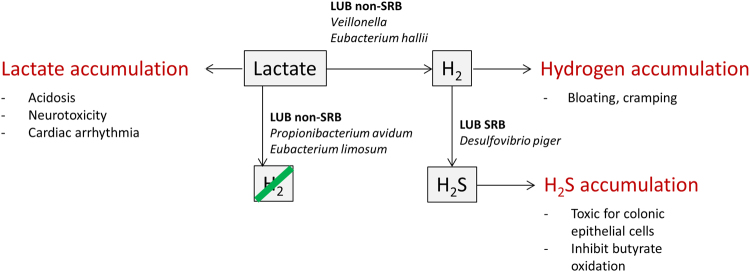
Schematic overview of lactate, H_2_, and H_2_S metabolism in infant gut.

Our findings also revealed an intricate lactate metabolism in the infant gut, involving production and utilization of lactate and H_2_, and eventually resulting in the accumulation of H_2_S. The interplay between these metabolites in the infant gut and their consequences for health and disease is illustrated in Fig. 6. While lactate accumulation could lead to acidosis, neurotoxicity, and cardiac arrhythmia, lactate utilization by LUB non-SRB may lead to excess H_2_ production, which is responsible for bloating and cramping in early life^22^. Unexpectedly, our data suggest the beneficial role of SRB as important H_2_ utilizers in infants. On the other hand, the detrimental effect of SRB on health and disease has been well studied, but only in the adult population. SRB reduce sulfate to H_2_S, which is toxic for colonic epithelial cells and can selectively inhibit butyrate oxidation *in vitro* ^44^. *In vivo*, high H_2_S and SRB levels have been shown in patients with IBD^24^ and to a lesser extent in patients with colorectal cancer^45^. We suggest that the presence of SRB in infants is beneficial as H_2_-utilizers, assuming that H_2_S production does not exceed the detoxification capacity of infant colonic epithelial cells. It should also be taken into account that infants, like adults, may have different sensitivities and thresholds to pain, with possibly different H_2_S detoxification capacities. Therefore, the impact of H_2_ or H_2_S on the host might vary between individuals.

Our *in vivo* data highlighted a complex lactate metabolism in the infant gut, and correlations between LPB and LUB. To investigate the interactions among the dominant LUB that modulate metabolic activity in the gut, we used an *in vitro* culture-based approach. The isolation of LUB from healthy infants provided an opportunity for physiologic and metabolic characterization of important key players. Anaerobic cultivation using Hungate tube methodology is suitable for cultivating strict anaerobes under highly controlled conditions independently of the metabolite absorption that occurs *in vivo* in the host intestine. However, one well-known drawback of culture dependent method is the lack of knowledge on growth requirement. In this study, our isolation medium (lactate YCFA) might not support the selection and isolation of *E. hallii* and *D. piger* among other lactate-utilizers. Interestingly, the characterization of growth and metabolic activity of five LUB strains representing the dominant LUB groups in the infant gut in four media revealed five distinct metabolic profiles, indicating the metabolic diversity of strains within the functional bacterial group sharing the same function of lactate utilization. Understanding factors of the metabolic balance that can result in gastrointestinal symptoms may allow specific modulation of bacterial activity. Among the tested strains, *V. ratti* produced the highest amount of H_2_. This finding, together with the high prevalence and level of *Veillonella* in infants^26^, supports our hypothesis that increased H_2_ production by LUB might result in acute H_2_ accumulation, potentially leading to crying and IC.

In this study, we used the anaerobic Hungate technique to investigate interactions between *V. ratti*, *E. limosum*, and *D. piger* strains. These strains represent three main lactate utilization pathways with different end products. In a recent Brazilian cohort study of 12 infants in the first year of life, *E. limosum* were detected by qPCR in fecal samples four infants, suggesting the early colonization of this genus^27^. *V. ratti* dominated the butyrate-producer *E. limosum* in this co-culture, which could be explained by the fast growth of *Veillonella* compared to *E. limosum* in lactate medium). Furthermore, the inability to ferment other carbohydrates makes *Veillonella* an efficient competitor for lactate. This result was in concordance to our previous finding, where *Veillonella* were identified as one of the key species in the infant gut^26^. Interestingly, although *E. limosum* was dominated by *V. ratti*, H_2_ concentration was decreased by 2 fold in co-culture compared with *V. ratti* single culture, indicating the potential of *E. limosum* to reduce H_2_ under the tested conditions.

Our HPLC and qPCR data suggested that *V. ratti* and *D. piger* co-existed in co-culture without one strain dominating the other. Interestingly, gas composition analysis showed a 25-fold depletion of H_2_ in *V. ratti* - *D. piger* co-culture compared to *V. ratti* single culture. This result could be explained by metabolic cross-feeding, where H_2_ produced from *V. ratti* serves as a substrate for *D. piger* ^39^. This result is consistent with our previous *in vivo* finding in which *Veillonella* and SRB were present at a high level and in high prevalence in the first 6 months of life^26^. It is noteworthy that this competition might change over time, where relative abundance of *Veillonella* gradually decreases^26^, whereas the prevalence of SRB increases across age^44, 46^. This shift could be explained by the ability of SRB to use host factors as substrates, such as mucin, which is increasingly produced over time^47^.

Despite many years of research, the debate on the etiology of IC has not reached consensus. This is reflected in the diverse and controversial therapeutic means. There is weak evidence to support the use of conventional pharmaceutical products (simethicone, dicyclomine, hydrochloride, and cimetropium bromide) or behavioral interventions (counselling, car ride simulation, music) for IC treatment^48^. Dietary interventions focusing on the role of human milk and cow milk allergy are one of the most common treatment approaches. However, management of IC by food elimination may increase the potential risk of developing an IgE-mediated food allergy such as cow milk allergy which could be life-threatening^49^. The limited number of intervention studies using probiotics, including the most studied *Lactobacillus reuteri* DSM 17938, showed inconclusive effects^29^, suggesting that probiotics cannot be routinely recommended for treatment of IC^29, 50^. The potential mechanism of *Lactobacillus* as probiotic to improve colic symptoms is based on its antimicrobial activity against gas- forming coliforms whose concentration was higher in colicky infants than in healthy controls^41, 51, 52^. However, *L. reuteri*, a heterofermentative species of lactic acid bacteria, produces lactate as a main primary metabolite that can feed other gas-forming LUB.

One possible therapeutic approach involves using probiotic LUB that produce no or small amounts of H_2_ to compete with high H_2_-producing LUB such as *Veillonella*. We isolated butyrate-producing *E. limosum* and propionate-producing *P. avidum* from healthy infant feces and demonstrated the ability of *E. limosum* to reduce H_2_ in co-culture with *Veillonella*. In the future, the sequence of establishment of *E. limosum* and *P. avidum* and their possible role in alleviating colic symptoms should be investigated. At the same time, it is important to develop methods to enhance their colonization in the infant gut, e.g. by using prebiotics. A strategy to combat H_2_ accumulation in the colon could also aim to increase H_2_ utilization by hydrogenotrophs (methanogens, SRB, and reductive acetogens). However, no methane production has been reported below the age of three years^53, 54^, suggesting that the infant gut might not be a niche for methanogens. On the other hand, H_2_S can be toxic for colonic cells and inhibit butyrate oxidation in the cells^55^. In contrast, not much is known about acetogens in newborns and infants. Hence, future studies should investigate the colonization of acetogens and its potential as hydrogenotrophs in infants.

Because therapeutic strategies are specific for different types of microbial dysbiosis, it may be important to distinguish colicky infants suffering from lactate, H_2_ or H_2_S accumulation. Determination of such biomarkers, preferably in combination, should be accessed and integrated in further studies of IC. However, H_2_ production in respiration chamber is difficult to measure with young infants for ethical and practical reasons. Also the monitoring of breath H_2_ at the specific period of crying was not practical with our study design. Furthermore, the use of hydrogenase genes or and activity as biomarker of H_2_ metabolism may not be accurate due to the diversity and widespread of H_2_ metabolism among human gut microbes, with 71% of sequenced genomes encoding these enzymes^56^. To overcome these limitations in H_2_ measurement and confirm the microbial mechanism of infant colic, we are currently studying gnotobiotic rats colonized with infant microbiota from colic and non-colic infants.

In conclusion, our results found higher lactate-utilizing, H_2_-producing bacteria in crying and colicky infants, which suggests that acute accumulation of H_2_ plays a role in the etiology of colic. We characterized important lactate-utilizing key players in the infant gut, and highlighted the potential of alternative therapeutics using tailored probiotics, although further proof of concept in clinical trials are required. We emphasize that the balance between lactate-utilizing, H_2_-producing bacteria (e.g. *Veillonella*, *E. hallii*) and lactate-utilizing H_2_-utilizing bacteria (e.g. SRB) is key to infant gut health.

## Methods

### Study Design

We recruited a total of 40 healthy, term infants. Inclusion criteria were as follows: a term delivery (gestation period of 37–42 weeks), normal birth weight (female: 2.7–5.0 kg; male: 2.9–5.2 kg), no known gastroenterological or immunological disease of the mother, no congenital diseases of the infant (e.g. gastrointestinal abnormality or immune deficiency). We obtained written informed consent from mothers-to-be on behalf of their infants. The study protocol was approved by the Ethic Committee of ETH Zurich (Zurich, Switzerland) (Project EK 2012-N-36; date of approval 28.09.2012)^26^ and carried out in accordance with the relevant guidelines and regulations.

We designed a questionnaire to gain information regarding the infant gastrointestinal symptoms. At each time point, mothers were asked in person to rank the degree of bloating/flatulence and stomach cramping at four levels (no, light, medium, and strong) and to record the crying time (hours per day). IC were diagnosed according to Rome III Criteria (Rome III IC), in which the infant must include all of the following from birth to 4 months of age: i, paroxysms of irritability, fussing, or crying that start and stop without obvious cause; ii, episodes lasting 3 or more hours per day and occurring at least 3 days per week for at least 1 week; iii, no failure to thrive^30^ (Table 1).

### Sample collection

Fresh infant fecal samples were collected at 2 weeks, 1 month, 3 months, and 6 months of life. Samples were transported within 8 h at 4 °C under anaerobiosis until processing at the laboratory. Fecal aliquots were immediately cultured, while further aliquots were stored at −80 °C prior to DNA extraction for qPCR and Illumina Miseq sequencing.

### Enumeration of lactate-utilizing bacteria

Liquid media were boiled, flushed with 100% O_2_-free CO_2_, dispensed into CO_2_-flushed Hungate tubes, sealed with butyl rubber septa (Bellco Glass, Vineland, USA) and autoclaved before use. The total anaerobes, LUB-SRB, and LUB non-SRB communities were enumerated using the most probable number estimation as described earlier^26^.

### Isolation of lactate-utilizing bacteria

The human fecal LUB strains were isolated by the anaerobic roll tube method^57^ using molten M2GSC 2% agar^58^ containing 35 mM DL-lactate as sole carbon source. Serial 10-fold dilutions were prepared from 1 g of fresh feces and inoculated into M2GSC 35 mM DL-lactate medium. After incubation at 37 °C for 5 days, the concentration of the remaining lactate was determined enzymatically (Megazyme, Bray, Co. Wicklow, Ireland). Tubes with a final lactate concentration below 25 mM (lactate consumption of at least 10 mM) were selected for isolation from which 0.3 ml was inoculated into roll tubes in duplicate. After incubating for 5 days at 37 °C, isolated colonies were inoculated into the same liquid medium and incubated for 2 days. Isolates with an optical density (OD_600_) > 0.3 were further purified by second and third passage on roll tubes. Purity and morphology were assessed by gram-stain. Cells from pure cultures were harvested for DNA extraction, followed by 16 S rRNA genetic sequencing for taxonomical identification as described previously^13^.

### Characterization and interaction of LUB strains

Three LUB isolates from infant fecal samples (*V. ratti*, *P. avidum*, and *E. limosum*) and two strains from DSMZ (Deutsche Sammlung von Mikroorganismen und Zellkulturen GmbH, Braunschweig, Germany) (*E. hallii* - DSM 3353 and *D. piger* - DSM 749) were grown in triplicate in YCFA medium supplemented with 6 g/l lactose, 60 mM L-lactate, 60 mM DL-lactate, or 6 g/l glucose. YCFA is a medium widely used to cultivate and isolate fecal strict anaerobes^59^. Strict anaerobic conditions were used for culturing using the Hungate technique^60^, as described above.

*E. limosum* and *V. ratti*, isolated from infant fecal samples, and *D. piger* (DSM 749) were activated from frozen cultures, and sub-cultured every 24 h by inoculating 3% culture into 10 ml fresh YCFA media supplemented with 60 mM L-lactate. Single cultures and co-cultures of *E. limosum* and *V. ratti*, *E. limosum* and *D. piger*, and *V. ratti* and *D. piger* were performed in Hungate tubes containing YCFA-L-Lactate medium. For each measurement point, individual tubes were inoculated in triplicate with 0.3 ml of overnight cultures at an OD_600_ of 1.

For all tested cultures, OD_600_ of single and co-culture were measured at 0, 8, 24, and 48 h after inoculation. Metabolite concentrations were measured in culture supernatant at 48 h using HPLC analysis. Data were averaged from two independent experiments.

### Metabolite analysis

Lactose, glucose, lactate, and SCFA (acetate, propionate, and butyrate) were determined in fecal water as well as culture supernatant by using HPLC as previously described^26^. A volume of 0.15 mL of gas was collected from the headspace of the Hungate tube with a gas-tight syringe (Hamilton, model 1725/RN 250 mL, Fisher Scientific AG, Wohlen, Switzerland), and its H_2_ concentration was analyzed with a gas chromatograph (model 6890 N, Agilent Technologies, Santa Clara, CA, USA) equipped with a Porapak Q column (80/100; 166 mesh; Fluka Chemie AG, Buchs, Switzerland) and a flame ionization detector operated at 250 °C.

### DNA extraction

Two hundred milligrams of infant fecal samples were used for total DNA extraction with the FastDNA SPIN kit for Soil (MP Biomedicals, Illkirch, France) using the manufacturer’s instructions. DNA concentration was measured by absorbance at 260 nm using a NanoDrop® ND-1000 Spectrophotometer (Witec AG, Littau, Switzerland). DNA samples were stored at −20 °C prior to qPCR and MiSeq sequencing analyses.

### Quantitative PCR Analysis

One microliter of template genomic DNA was mixed with 2 x Kapa Sybr Fast qPCR Mastermix (Biolabo Scientifics Instruments, Châtel-St-Denis, Switzerland) in a total volume of 25 µl in a 96-well plate. The reactions were carried out in an ABI PRISM 7500-PCR sequence detection system (Applied Biosystems, Zug, Switzerland) as described previously^26, 61^. Specific primers targeting bacterial species prevalent in the infant gut microbiota and functional genes involved in lactate and H_2_ metabolism were described previously^26^. A series of tenfold diluted standard was included in each run. Standards were generated as previously described^61^.

### Amplicon sequencing

The microbiota community was analysed in fecal samples from a subgroup of 16 infants (8 colicky and 8 randomly-selected non-colicky infants). The sample preparation and sequencing were carried out at Microsynth AG (Balgach, Switzerland) as described in previous work^26^.

### Statistical analysis

Statistical analyses were carried out with IBM SPSS Statistics 20.0 (IBM SPSS Inc, Chicago, IL, USA). Cultural and qPCR data were log_10_ transformed, tested for normal distribution using Shapiro-Wilk test, and expressed as mean ± standard deviation (SD). For qPCR data, a default value of ½ the detection limit was assigned for values below the detection limit of the method. Means stratified by crying time and colic were compared pairwise using Student’s t-test for normally distributed data. A non-parametric Mann-Whitney test was performed when data were not normally distributed. *P* values < 0.05 were considered significant.

Spearman correlation R and corresponding *q* values between crying hours, flatulence, stomach cramps and colic criteria were calculated. Correlation graph were made using the R software (http://www.r-project.org).

## Electronic supplementary material

Supplementary Information
