# Supplementary material for: Lactate-utilizing community is associated with gut microbiota dysbiosis in colicky infants
Source: Sci Rep. 2017 Sep 11;7:11176. doi: 10.1038/s41598-017-11509-1 (PMC5593888; doi:10.1038/s41598-017-11509-1)
Supplement: Supplementary file 1 — Supplementary Information [file 41598_2017_11509_MOESM1_ESM.docx]

# Supplementary Information

**Lactate-utilizing community is associated with gut microbiota dysbiosis in colicky infants**

Van T. Pham, Christophe Lacroix, Christian P. Braegger, Christophe Chassard

**
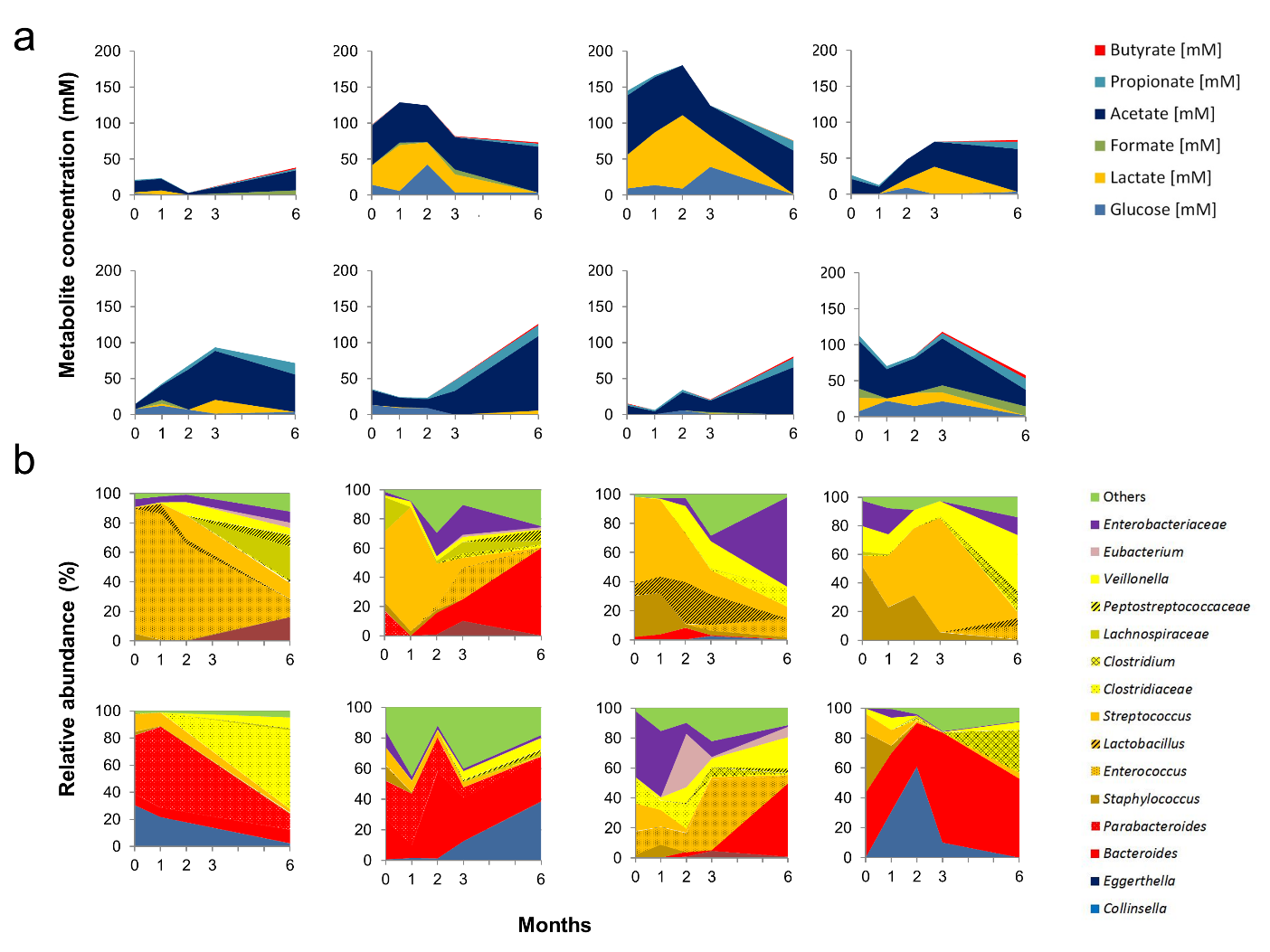
**

**Figure S1**. **(a)** Stacked metabolites concentration in fecal samples of 8 colicky infants using HPLC. **(b)** Relative abundance (%) of 16S rRNA genes at genus level analysed in fecal samples of 8 colicky infants using Illumina MiSeq. Values < 1% are summarized in the group “Others”.


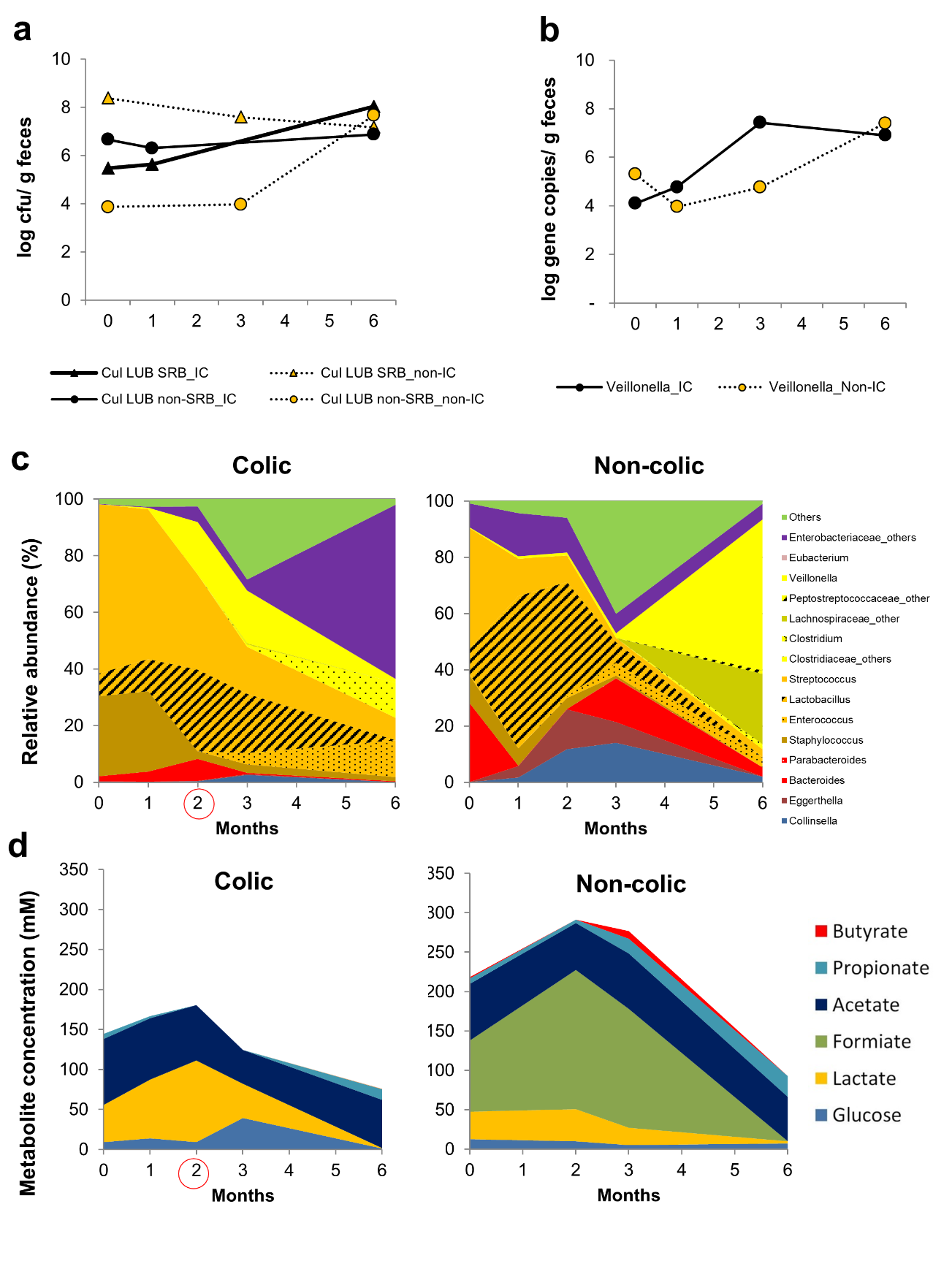


**Figure S2**. The gut microbiota and metabolite composition in fecal samples of a pair of twins. **(a)** Total counts of LUB SRB, LUB non-SRB in feces of colic (n=1; closed symbols) and non-colic (n=1; open symbols) infants at 2 weeks, 1 month, 3, and 6 months. Values are expressed as means ± SD log cfu/ g feces. **(b)** *Veillonella* levels between colicky (closed symbols) and non-colicky (open symbols) infants at 2 weeks, 1 month, 3, and 6 months. Values are expressed as means ± SD log gene copies/g feces. **(c)** Relative abundance (%) of 16S rRNA genes at genus level analysed in fecal samples of colicky and non-colicky infants using Illumina MiSeq. Values < 1% are summarized in the group “Others”. **(d)** Stacked metabolites concentration in fecal samples of colicky and non-colicky infants using HPLC. Red circle indicate the time point when colic was diagnosed.

**Figure S3**. Production of H_2_ by LUB isolated from infant feces (*E. limosum*, *P. avidum*, and *V. ratti*) and culture collection LUB strains (*D. piger* DSM 749 and *E. hallii* DSM 3353) after 48 h incubation in YCFA medium supplied with DL-Lactate.

**
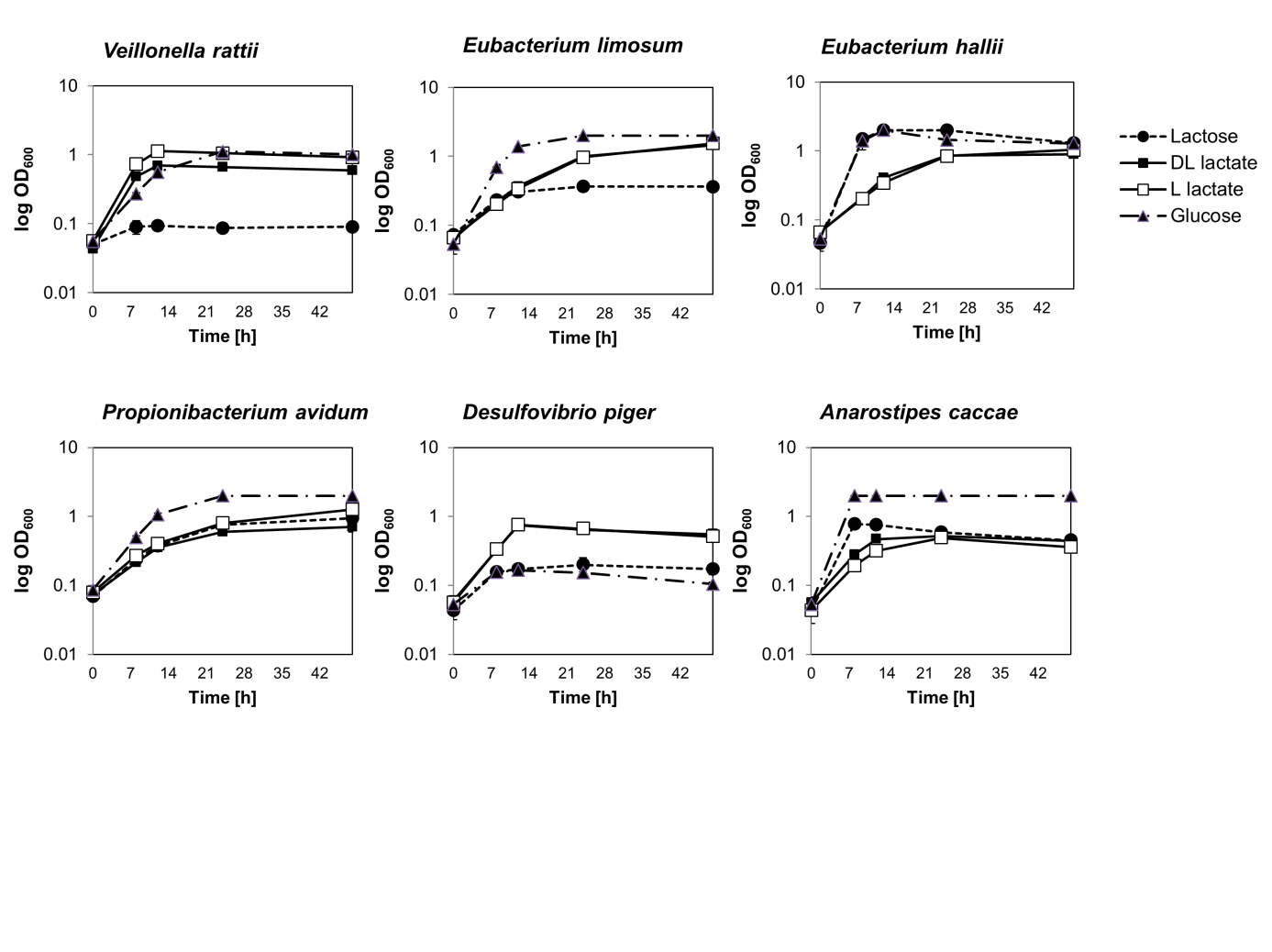
**

**Figure S4.** Log OD_600_ of LUB isolated from infant feces (*E. limosum*, *P. avidum*, and *V. ratti*) and culture collection LUB strains (*D. piger* DSM 749 and *E. hallii* DSM 3353) after 48h incubation in YCFA medium supplemented with lactose, DL-lactate, L-lactate or glucose as sole carbon source.


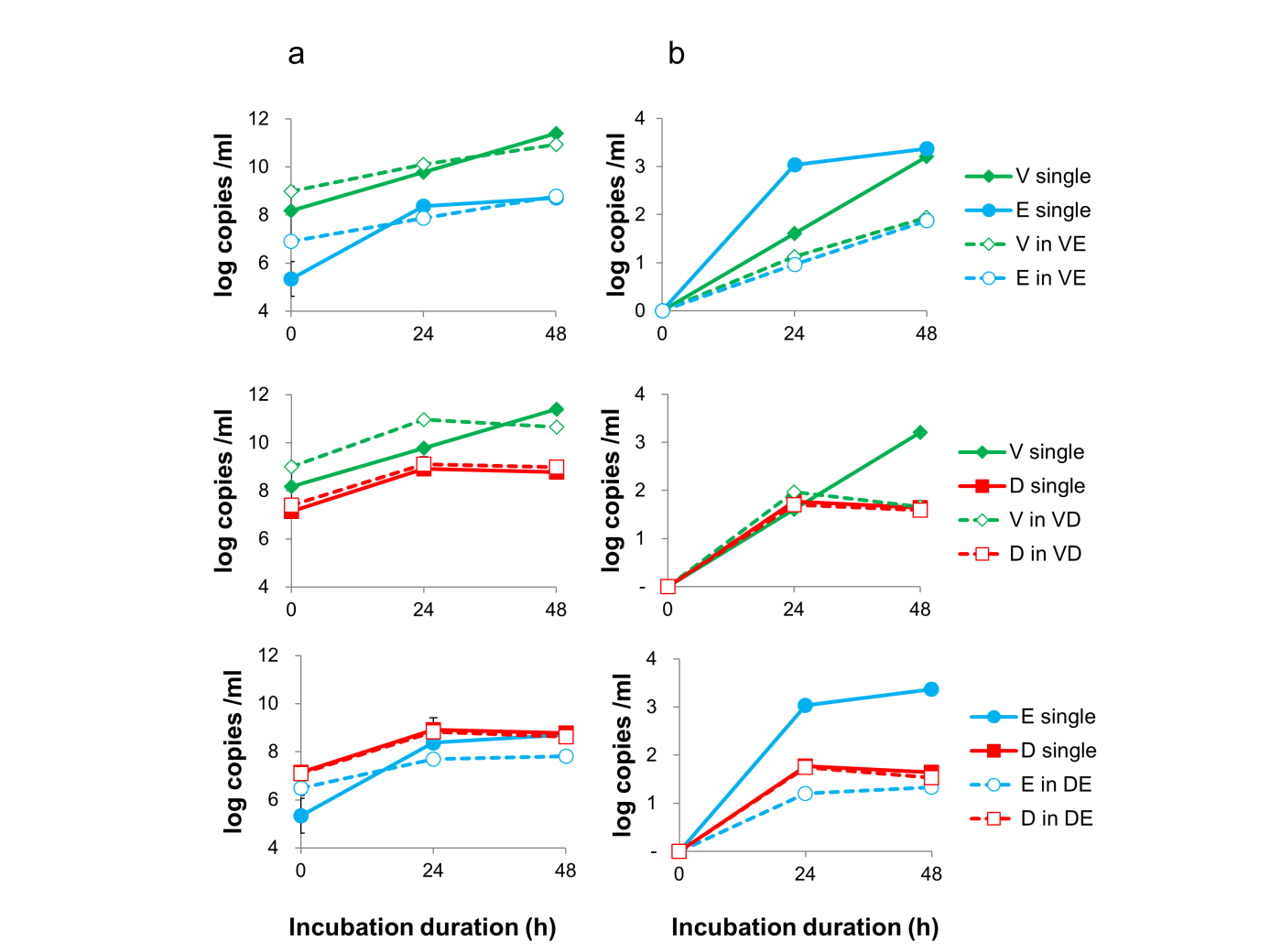


**Figure S5 (a)** Log gene copies of *V. ratti* (V)*, E. limosum* (E) and *D. piger* (D) (DSM 749) in single and co-cultures enumerated by qPCR. **(b)** Log increase of *V. ratti, E. limosum* and *D. piger* levels in single and co-cultures.

*V. ratti* and *E. limosum* strains were isolated from infant feces (Pham 2016).

**Table S1.** Comparison of fecal microbial levels of (**a**) non-colicky and colicky infants; and (**b**) of infants who cried <1h/d and infants who cried >1h/d. Values obtained by qPCR are expressed as means (SD) log gene copies/g feces. Values obtained by cultural method are expressed as means (SD) log cfu/ g feces. N.S, not significant (*P* > 0.05)

| a |  |  |  |  | |  | |  | |  | |  | | |  |  | | |  |
| --- | --- | --- | --- | --- | --- | --- | --- | --- | --- | --- | --- | --- | --- | --- | --- | --- | --- | --- | --- |
|  | **2 weeks** | | | **1 month** | | | | | **3 months** | | | | | **6 months** | | | | | |
|  | non-colic | colic | *P* | non-colic | colic | | *P* | | non-colic | | colic | | *P* | non-colic | | | colic | *P* | |
| Total bacteria | 11.65 (0.48) | 11.69 (0.35) | N.S | 11.12 (0.92) | 11.31 (0.65) | | N.S | | 10.76 (1.28) | | 10.57 (0.93) | | N.S | 11.13 (2.03) | | | 10.86 (1.08) | N.S | |
| *Eubacterium hallii* | 4.16 (0.49) | 5.39 (1.23) | 0.03 | 4.13 (0.38) | 5.25 (1.59) | | N.S | | 4.41 (1.03) | | 4.50 (0.72) | | N.S | 4.18 (0.52) | | | 4.03 (0.14) | N.S | |
| SRB | 3.10 (1.47) | 2.35 (0.05) | N.S | 2.86 (1.21) | 2.92 (0.79) | | N.S | | 2.48 (0.54) | | 2.45 (0.46) | | N.S | 2.49 (0.68) | | | 2.65 (0.69) | N.S | |
| *Veillonella* | 7.57 (1.66) | 6.88 (1.76) | N.S | 7.04 (1.41) | 7.05 (1.36) | | N.S | | 6.90 (1.81) | | 6.89 (1.46) | | N.S | 8.35 (0.98) | | | 8.00 (1.19) | N.S | |
| *Lactobacillus* | 6.56 (1.52) | 6.67 (1.38) | N.S | 5.73 (1.42) | 6.13 (1.74) | | N.S | | 5.77 (1.35) | | 5.95 (1.42) | | N.S | 6.31 (1.64) | | | 6.24 (1.09) | N.S | |
| *Bifidobacterium* | 9.41 (1.75) | 10.16(1.49) | N.S | 9.09 (1.62) | 10.01(0.60) | | 0.02 | | 8.80 (1.66) | | 9.07 (0.68) | | N.S | 8.43 (1.30) | | | 8.54 (1.55) | N.S | |
| *Roseburia* | 5.31 (0.31) | 5.17 (0.50) | N.S | 5.35 (0.45) | 5.32 (0.30) | | 0.01 | | 5.04 (0.72) | | 5.27 (0.14) | | N.S | 5.12 (0.94) | | | 4.84 (0.77) | N.S | |
| Clostridium cluster IV | 6.31 (0.68) | 6.17 (0.38) | N.S | 6.31 (0.71) | 5.43 (0.86) | | N.S | | 6.09 (0.61) | | 5.58 (0.54) | | N.S | 6.23 (0.98) | | | 6.09 (0.84) | N.S | |
| *Enterobacteriaceae* | 8.07 (1.73) | 7.69 (1.05) | N.S | 7.59 (1.82) | 6.98 (1.14) | | N.S | | 7.35 (1.89) | | 7.27 (1.4) | | N.S | 7.91 (1.22) | | | 7.94 (0.92) | N.S | |
| *Streptococcus* | 8.68 (0.97) | 9.01 (0.78) | N.S | 7.96 (0.88) | 8.18 (0.89) | | N.S | | 7.75 (0.58) | | 7.73 (0.8) | | N.S | 7.40 (0.92) | | | 7.37 (0.35) | N.S | |
| *Staphylococcus* | 8.36 (0.82) | 8.30 (0.81) | N.S | 7.65 (0.9) | 7.54 (0.54) | | N.S | | 7.38 (0.38) | | 7.46 (0.27) | | N.S | 7.33 (0.66) | | | 7.34 (0.29) | N.S | |
| *Bacteroides* | 7.58 (2.06) | 7.93 (1.98) | N.S | 7.20 (1.94) | 7.18 (1.87) | | N.S | | 7.47 (1.75) | | 7.64 (1.09) | | N.S | 8.29 (2.15) | | | 8.41 (1.85) | N.S | |
| Firmicutes | 9.66 (0.66) | 9.69 (0.37) | N.S | 9.09 (0.97) | 9.28 (0.51) | | N.S | | 8.56 (1.45) | | 8.68 (0.93) | | N.S | 9.14 (0.91) | | | 9.28 (0.77) | N.S | |
| *Faecalibacterium prausnitzii* | 6.34 (0.24) | 6.29 (0.17) | N.S | 6.42 (0.74) | 6.33 (0.35) | | N.S | | 6.30 (0.17) | | 6.24 (0.39) | | N.S | 6.41 (1.10) | | | 6.82 (1.04) | N.S | |
| Culture total anaerobes | 9.88 (0.56) | 10.14 (0.45) | N.S | 10.01 (0.56) | 10.00 (0.45) | | N.S | | 10.17 (0.51) | | 10.36 (0.62) | | N.S | 10.07 (0.40) | | | 10.14 (0.28) | N.S | |
| Culture LUB SRB | 6.36 (1.14) | 6.24 (0.82) | N.S | 6.37 (1.28) | 6.26 (0.87) | | N.S | | 6.91 (0.83) | | 7.02 (0.94) | | N.S | 7.18 (0.54) | | | 7.10 (0.72) | N.S | |
| Culture LUB non SRB | 5.69 (1.64) | 6.13 (1.43) | N.S | 5.53 (1.45) | 5.96 (1.09) | | N.S | | 5.95 (2.09) | | 6.98 (1.42) | | N.S | 7.24 (1.30) | | | 7.09 (1.01) | N.S | |
| b |  | | |  | | | | |  | | | | |  | | | | | |
|  | **2 weeks** | | | **1 month** | | | | | **3 months** | | | | | **6 months** | | | | | |
|  | <1h/d | >1h/d | P | <1h/d | >1h/d | | P | | <1h/d | | >1h/d | | P | <1h/d | | | >1h/d | P | |
| Total bacteria | 11.71 (0.52) | 11.67(0.27) | N.S | 11.05 (0.87) | 11.25 (0.85) | | N.S | | 10.63 (1.35) | | 10.63 (1.15) | | N.S | 11.28 (2.11) | | | 10.56 (0.93) | N.S | |
| *Eubacterium hallii* | 4.49 (0.93) | 4.39(0.85) | N.S | 4.46 (1.02) | 4.35 (0.89) | | N.S | | 4.54 (1.18) | | 4.35 (0.84) | | N.S | 4.22 (0.53) | | | 3.95 (0.13) | 0.022 | |
| SRB | 2.90 (1.41) | 2.95(1.39) | N.S | 3.03 (1.5) | 2.78 (1.04) | | N.S | | 2.59 (0.71) | | 2.41 (0.31) | | N.S | 2.56 (0.64) | | | 2.43 (0.80) | N.S | |
| *Veillonella* | 7.21 (1.96) | 7.46(1.45) | N.S | 6.81 (0.94) | 7.20 (1.46) | | N.S | | 6.65 (1.64) | | 7.09 (1.82) | | N.S | 8.43 (1.07) | | | 7.90 (0.79) | N.S | |
| *Lactobacillus* | 6.94 (1.93) | 6.34(1.09) | N.S | 5.25 (1.58) | 6.03 (1.46) | | N.S | | 5.44 (0.97) | | 5.77 (1.34) | | N.S | 6.36 (1.54) | | | 6.11 (1.55) | N.S | |
| *Bifidobacterium* | 9.54 (1.61) | 9.8(1.74) | N.S | 9.04 (1.84) | 9.42 (1.36) | | N.S | | 8.38 (1.54) | | 9.06 (1.53) | | N.S | 8.73 (1.21) | | | 7.74 (1.42) | 0.037 | |
| *Roseburia* | 5.25 (0.42) | 5.29(0.32) | N.S | 5.23 (0.11) | 5.39 (0.48) | | N.S | | 4.90 (0.84) | | 5.22 (0.5) | | N.S | 5.07 (1.05) | | | 5.05 (0.40) | N.S | |
| Clostridium cluster IV | 6.3 (0.83) | 6.26(0.46) | N.S | 6.42 (1.27) | 6.03 (0.64) | | N.S | | 6.01 (0.75) | | 5.99 (0.51) | | N.S | 6.15 (0.95) | | | 6.34 (0.96) | N.S | |
| *Enterobacteriaceae* | 8.30 (1.74) | 7.91(1.49) | N.S | 7.65 (2.32) | 7.42 (1.56) | | N.S | | 7.47 (1.98) | | 6.94 (1.63) | | N.S | 8.14 (1.03) | | | 7.34 (1.3) | 0.05 | |
| *Streptococcus* | 8.84 (1.08) | 8.7(0.83) | N.S | 7.72 (0.6) | 8.11 (0.94) | | N.S | | 7.80 (0.72) | | 7.67 (0.55) | | N.S | 7.32 (0.78) | | | 7.60 (0.95) | N.S | |
| *Staphylococcus* | 8.47 (0.86) | 8.27(0.78) | N.S | 7.47 (0.74) | 7.71 (0.89) | | N.S | | 7.44 (0.31) | | 7.40 (0.38) | | N.S | 7.30 (0.34) | | | 7.41 (1.03) | N.S | |
| *Bacteroides* | 8.11 (1.91) | 7.4(2.13) | N.S | 7.13 (2.37) | 7.27 (1.83) | | N.S | | 7.86 (1.36) | | 6.95 (1.78) | | N.S | 8.32 (2.16) | | | 8.28 (1.91) | N.S | |
| Firmicutes | 9.69 (0.72) | 9.71(0.39) | N.S | 8.90 (0.93) | 9.26 (0.83) | | N.S | | 8.26 (1.47) | | 8.66 (1.24) | | N.S | 9.25 (0.83) | | | 8.98 (1.00) | N.S | |
| *Faecalibacterium prausnitzii* | 6.33 (0.28) | 6.36(0.07) | N.S | 6.62 (1.11) | 6.34 (0.5) | | N.S | | 6.29 (0.21) | | 6.30 (0.2) | | N.S | 6.43 (0.92) | | | 6.66 (1.47) | N.S | |
| Culture total anaerobes | 10.05 (0.53) | 9.88(0.54) | N.S | 10.02 (0.64) | 9.97 (0.51) | | N.S | | 10.21 (0.6) | | 10.22 (0.49) | | N.S | 10.06 (0.41) | | | 10.12 (0.29) | N.S | |
| Culture LUB SRB | 6.69 (1.19) | 6.04(0.92) | N.S | 6.63 (0.82) | 6.22 (1.31) | | N.S | | 7.05 (0.76) | | 6.79 (0.92) | | N.S | 7.18 (0.61) | | | 7.14 (0.48) | N.S | |
| Culture LUB non SRB | 5.69 (1.64) | 5.78(1.55) | N.S | 5.25 (1.48) | 5.82 (1.37) | | N.S | | 5.43 (1.85) | | 6.72 (2.05) | | N.S | 7.13 (1.27) | | | 7.43 (1.15) | N.S | |

**Table S2.** Fecal metabolite concentrations of non-colicky and colicky infants.

| Metabolite conc. (mM) | 2 weeks | | |  | 1 month | | |  | 2 months | | |  | 3 months | | |  | 6 months | | |
| --- | --- | --- | --- | --- | --- | --- | --- | --- | --- | --- | --- | --- | --- | --- | --- | --- | --- | --- | --- |
|  | Non-colic | Colic | *P* |  | Non-colic | Colic | *P* |  | Non-colic | Colic | *P* |  | Non-colic | Colic | *P* |  | Non-colic | Colic | *P* |
|  | n=31 | n=8 |  |  | n=28 | n=8 |  |  | n=30 | n=8 |  |  | n=29 | n=8 |  |  | n=32 | n=8 |  |
| Glucose | 9.06 (11.91) | 6.86 (5.52) | N.S |  | 8.03 (11.58) | 9.61 (10.07) | N.S |  | 5.58 (6.69) | 9.53 (13.93) | N.S |  | 7.72 (10.57) | 7.2 (14.23) | N.S |  | 7.07 (13.41) | 1.19 (1.69) | N.S |
| Lactate | 12.02 (19.05) | 10.85 (17.16) | N.S |  | 11.46 (13.47) | 18.41 (31.03) | N.S |  | 15.53 (21.29) | 17.81 (35.62) | N.S |  | 22.97 (31.9) | 12.85 (16.81) | N.S |  | 13.81 (24.92) | 5.97 (11.5) | N.S |
| Formate | 4.02 (16.24) | 0.00 (0.00) | N.S |  | 1.2 (3.65) | 1.11 (2.12) | N.S |  | 18.15 (45.67) | 0.00 (0.00) | N.S |  | 14.74 (36.82) | 1.26 (2.57) | N.S |  | 2.98 (7.86) | 0.84 (2.38) | N.S |
| Acetate | 30.9 (23.14) | 32.02 (27.96) | N.S |  | 37.54 (21.15) | 39.24 (30.05) | N.S |  | 38.92 (24.92) | 36.86 (24.86) | N.S |  | 50.07 (34.09) | 40.89 (21.71) | N.S |  | 56.63 (28.29) | 64.07 (21.66) | N.S |
| Propionate | 4.51 (5.54) | 2.30 (2.28) | N.S |  | 5.97 (9.3) | 2.41 (2.16) | N.S |  | 4.82 (4.71) | 3.29 (4.79) | N.S |  | 6.29 (6.77) | 5.60 (6.56) | N.S |  | 14.46 (11.03) | 9.74 (5.56) | N.S |
| Butyrate | 1.29 (2.00) | 0.57 (1.09) | N.S |  | 1.00 (1.36) | 1.13 (3.20) | N.S |  | 1.81 (2.59) | 0.68 (1.38) | N.S |  | 1.56 (2.70) | 0.61 (0.82) | N.S |  | 3.18 (3.16) | 1.76 (1.34) | N.S |

Values obtained by HPLC are expressed as means (SD). N.S, not significant (*P* > 0.05)
